# Exploration of the substances and key processing steps related to the sweetness of Niangniang tea

**DOI:** 10.1016/j.fochx.2025.102556

**Published:** 2025-05-15

**Authors:** Qi Wei, Xiaobo Liu, Shanshan Long, Luqin Zhu, Hongzhiyuan Yang, Xue Song, Yishu Peng, Taolin Chen, Jianjun Liu, Beibei Wen, Meifeng Li

**Affiliations:** a The College of Tea Science at Guizhou University*,* Guiyang 550000*,* China; b Tea Research Institute*,* Zhejiang University*,* Hangzhou 310058*,* China

**Keywords:** Niangniang tea, Sweetness, Metabolome, Key substances, Key processing step

## Abstract

Niangniang tea (NNT) is processed using a unique twice bunch-drying method, which tastes very sweet. In order to explore the key substances related to the sweetness of NNT, we processed green tea (GT), yellow tea (YT) and NNT and conducted a sensory evaluation and metabolomic analysis. NNT was the sweetest, with the lowest contents of total polyphenol and caffeine and highest content of soluble sugar. A total of 365 differential substances were identified in all the samples, most of which were flavonoids, alkaloids, and terpenoids. Thirty differential substances, especially caffeine, turanose, kojibiose, *L*-phenylalanine, L-leucine, and flavone, were explored as important factors contributing to sweetness. The second bunch-drying step was found to be critical to enhance the sweetness and reduce the bitterness of NNT. This study clarified the chemical basis for the sweetness of NNT and provides insights for the potential reduction of bitterness and astringency in summer and autumn tea.

## Highlights

• Niangniang (NNT) tea tastes very sweet compared to green tea and yellow tea.
• Thirty substances were considered to be important to the sweetness of NNT.
• The second bunch-drying was crucial to enhance sweet substances and reduce bitter substances.
• Caffeine, Turanose, Kojibiose, L-Phenylalanine, L-Leucine, and Flavone were key to affecting NNT sweetness.
• The twice bunching drying has great potential to improve the quality of summer and autumn tea.

## 1 Introduction

Tea is the most popular and consumed beverage after water. Various phytochemical compounds of tea contribute to the multiple health benefits and flavors, which are the most crucial and intuitive criteria for quality evaluation. The flavor characteristics, generally comprising bitterness, astringency, umami, and sweetness, are considered the four fundamental elements of the taste of different teas. Among them, sweetness is crucial as it reduces the bitterness and astringency, and softens the exciting flavor of tea.

Many chemicals can affect the sweetness of a tea infusion. Soluble sugars, which impart a fresh and sweet taste and relieve the bitterness and astringency, are crucial contributors to the sweetness of tea infusions (Wei, Luo, & Zeng, 2023). Amino acids elicit complex tastes and add layers of flavor to tea infusions. Alanine, glycine, serine, threonine, and proline are sweet amino acids; aspartic acid and glutamic acid are umami amino acids; and leucine, isoleucine, arginine, phenylalanine, valine, histidine, and methionine are bitter amino acids. Alkaloids, terpenoids, glycosides, polyphenols, and bitter amino acids contribute to the bitter and astringency taste of tea infusions. Catechin accounts for 70 %–80 % of the total polyphenols in tea, and ester-type catechins impart a stronger bitterness and astringency than non-ester types (Y.-N. Zhang et al., 2016). In tea infusions, amino acids and catechins can have synergistic, additive, or inhibitory effects on taste. Umami amino acids, such as theanine, glutamic acid (Glu), and aspartic acid (Asp), significantly reduce the bitterness of tea infusions through easy bonding on their reciprocal chemical structures (Z. Liu et al., 2023). This indicates that the sweetness of tea infusions is not only affected by the concentrations of sweet or bitter compounds, but by the interactions among different taste substances.

In order to improve the sweetness of tea infusions and thus enhance tea quality, various methods and techniques have been adopted. These include screening germplasm resources with low levels of tea polyphenols and caffeine, processing elite tea with early spring leaves, and improve the processing conditions at the stage of withering (Feng et al., 2014; Lee et al., 2014; Lin et al., 2022). Among them, optimizing the conditions of tea processing to improve the sweetness of the tea infusion is popular. Appropriately prolonging the withering time and red-light irradiation could promote many macromolecular compounds to hydrolyze into small molecules. The esterified catechins, polysaccharides, and proteins are hydrolyzed into non-esterified catechins, soluble sugars, and amino acids, which improve the sweetness of the tea infusion (Y. Wang, Zheng, et al., 2019). High-temperature fixing triggers the degradation of proteins and polysaccharides into free amino acids, soluble sugars, and other umami and sweet substances (Yu, Huang, et al., 2023). The “yellowing” process of yellow tea (YT) causes an increase in catechins (C), gallocatechin gallate (GCG), and theaflavins but a decline in epigallocatechin gallate (EGCG) and the ratio of tea polyphenols to amino acids, which reduces the bitterness and astringency of YT while increasing the sweetness (Fan et al., 2022). During the drying process of green tea (GT), most catechins and flavonols are decreased while total soluble sugars are increased, which enhances the sweetness and mellowness of the tea infusion by increasing the ratio of sweet substances to bitter substances (Ye et al., 2022). In summary, withering, yellowing, fixing, and drying can all improve the sweetness of tea infusions by changing the concentration of many taste compounds.

Niangniang tea (NNT) is processed from the landrace tea plant populations in Zhenfeng County, Guizhou Province. According to the tea classification (Xia, 2014), NNT is classified as a special green tea. NNT processing involves spreading, fixation, rolling, bunching (like a Chinese brush tip), and two steps of bunch-drying. NNT has significant sweet characteristics; however, it is still unknown whether the sweet taste is derived from metabolites or the unique processing. Furthermore, the mechanism underlying the formation of sweetness during the two bunch-drying steps is unknown. In this study, GT, YT and NNT were processed from fresh leaves from the landrace tea plant populations. Sensory evaluation, non-target metabolome detection, multivariate analysis, weighted correlation network analysis (WGCNA), and the current literature were used to explore the mechanism driving the formation of the sweetness of NNT.

## 2 Materials and methods

### 2.1 Materials and reagents

In July 2022, fresh tea leaves of the landrace tea plant populations were picked at Poliu Village, Longchang Town, and Zhenfeng County, Guizhou Province.

HPLC grade methanol and acetonitrile were purchased from ANPEL Laboratory Technologies (Shanghai, China). HPLC grade formic acid was purchased from Merck (Darmstadt, Germany).

### 2.2 Tea sample preparation

**Fig. 1:**
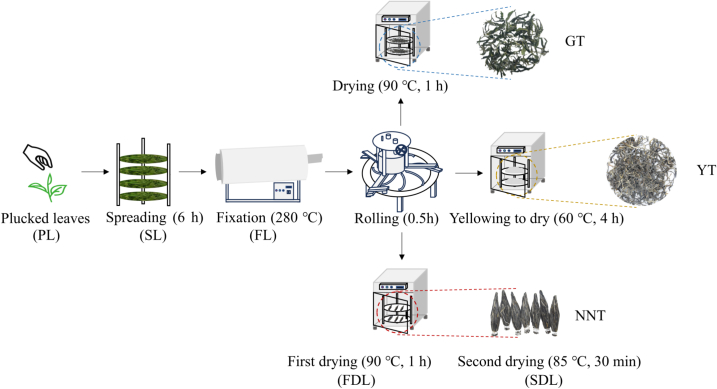
Processing flowchart for three types of tea.

GT, YT, and NNT (one bud, two to three leaves) were processed according to standard processing methods as showed in Fig. 1.

In order to better understand the dynamic evolution in substances of the three types of tea, samples were obtained after every important processing step. Put more concretely, samples were taken when leaves were plucked from stems (plucked leaves, PL), after spreading for 6 h (spread leaves, SL), after fixation (fixed leaves, FL), after the first drying (first dried leaves, FDL) and after the second drying (second dried leaves, SDL) (Fig. 1).

### 2.3 Quantitative descriptive analysis

A sensory evaluation was conducted in a room with a temperature of 23 ± 1 °C. The evaluation team consisted of 10 trained teachers and students (aged 23–40 years; four male and six female individuals) from Guizhou University. All members completed at least 60 h of descriptive analysis group training. Based on Herbert Stone's quantitative description analysis theory (Sidel et al., 2018), descriptive analysis was performed on five taste attributes: bitterness, umami, astringency, sweetness, and thickness. The number 1–5 indicates very weak, weak, moderate, strong, and very strong, respectively. Each sample was randomly evaluated three times and the average value was determined.

### 2.4 Nonvolatile compound analysis using ultra-high performance liquid chromatography mass spectrometry (UHPLC/MS)

#### 2.4.1 Sample preparation and metabolite extraction

The freeze-dried samples were crushed with a mixer mill for 240 s at 45 Hz. Ten milligram aliquots of the individual samples were precisely weighed and were transferred to an Eppendorf tube containing 500 μL extract solution (methanol/water 3:1, precooled to −40 °C, containing internal standard). After 30 s of vortexing, the samples were homogenized at 45 Hz for 4 min and sonicated for 5 min in an ice-water bath. Homogenization and sonication were repeated three times. Then the samples were extracted overnight at 4 °C on a shaker and centrifuged at 12,000 rpm (RCF = 13,800 (× *g*), *R* = 8.6 cm) for 15 min at 4 °C. The supernatant was carefully filtered through a 0.22 μm microporous membrane and the resulting supernatants were diluted 10× with a methanol/water mixture (v:v 3:1, containing internal standard), vortexed for 30 s, and transferred to 2 mL glass vials. Then, 20 μL was taken from each sample and pooled for quality control (QC) samples. Samples were stored at −80 °C until UHPLC-MS analysis.

#### 2.4.2 UHPLC-MS analysis

The UHPLC separation was carried out using an EXIONLC System (Sciex, MA, USA). The mobile phase A was 0.1 % formic acid in water and mobile phase B was acetonitrile. The column temperature was set at 40 °C. The autosampler temperature was set at 4 °C and the injection volume was 2 μL. A Sciex QTrap 6500+ (Sciex Technologies) was used for the assay development. Typical ion source parameters were: IonSpray voltage, +5500/−4500 V; curtain gas, 35 psi; temperature, 400 °C; ion source gas 1, 60 psi; and ion source gas 2, 60 psi, DP: ± 100 V.

#### 2.4.3 Data preprocessing and annotation

SCIEX Analyst Workstation Software (Version 1.6.3) was employed for MRM data acquisition and processing. MS raw data (.wiff) files were converted to the TXT format using MSconverter. An in-house R program and database were applied for peak detection and annotation (Z. Zhang et al., 2015).

### 2.5 Association of nonvolatile compounds and taste quality via WGCNA

A co-expression network was constructed using the R package WGCNA to study the relationship between taste properties (bitterness, umami, astringency, sweetness, and thickness) and the nonvolatile compounds. In this study, the correlation matrix soft-thresholding power β was 16, the topological overlap metric (TOM) was calculated using the adjacent matrix, and the tree diagram was drawn using the dissimilarity TOM. The dynamic tree cut algorithm was used to create modules and assign different colors. Highly related metabolites were clustered in one module.

### 2.6 Statistical analysis

All analyses were repeated three times. A Nightingale Rose chart was created using the OmicStudio tools at https://www.omicstudio.cn/tool. A correlation heat map was created using www.chiplot.online. Bar charts and line graphs were created using GraphPad.Prism.9.5. All other plots were created using the Metware Cloud, a free online platform for data analysis (https://cloud.metware.cn).

## 3 Results

### 3.1 Comparative sensory evaluation

**Fig. 2:**
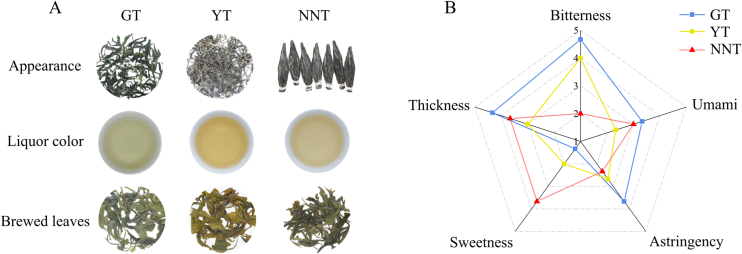
Comparative sensory evaluation. (A) Appearance, liquor color, and brewed leaves of three types of tea. (B) Radar map of five taste properties in three types of tea. Numbers indicate the intensity of the five taste attributes.

To determine whether the sweetness of NNT is due to the biochemical constituents of the landrace tea plant populations or the unique tea processing methods, NNT, GT, and YT were processed with a bud with three leaves and then submitted to quantitative descriptive analysis (Fig. 2A). NNT was the sweetest and the least bitter and astringent; GT had the strongest thickness, bitterness, umami and astringency, and was the least sweet; YT was moderate in bitterness, sweetness and astringency flavors, and had the weakest thickness and umami flavors (Fig. 2B). These results indicated that the unique NNT processing methods contributed to the sweetness of the NNT infusion.

### 3.2 Quality components in the three tea types

**Fig. 3:**
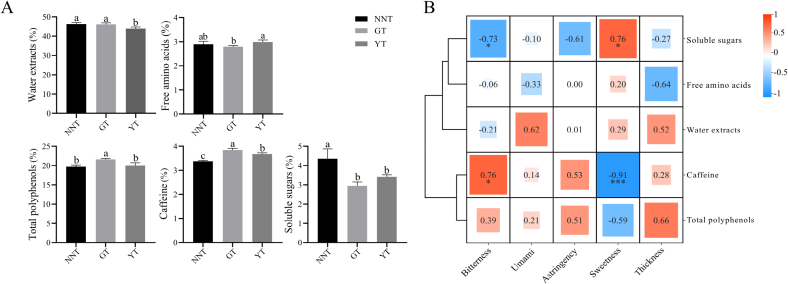
Analysis of main quality components. (A) Contents of the main quality components. (B) Heat map of the correlations between the main quality components and taste attributes. * significant and *** highly significant.

The contents of the main components contributing to the taste of the tea infusions were detected, including total polyphenols (TPP), free amino acids (FAA), caffeine, soluble sugar (SS), and water extracts (WE) (Fig. 3A). The content of SS was high in NNT but extremely low in GT. NNT had the least caffeine and GT had the highest caffeine content. The content of total polyphenols was significantly lower in NNT and YT compared to GT. The FAA content of NNT was between that of GT and YT.

In order to determine the contribution of the main quality components to the taste attributes of the three types of tea, the Pearson correlation coefficient (PCC) was used to explore correlations (PCC > 0.7, and *P*-value <0.05). SS had a significant and positive correlation with sweetness and a significant and negative correlation with bitterness. Caffeine had a significant and negative correlation with sweetness and a significant and positive correlation with bitterness. The total polyphenol content was negatively correlated with sweetness and positively correlated with bitterness, but the correlations were not significant (Fig. 3B). These results indicated that the higher SS and lower caffeine may be the key factors contributing to the sweetness of NNT.

### 3.3 Overall characterization of non-volatile metabolites

**Fig. 4:**
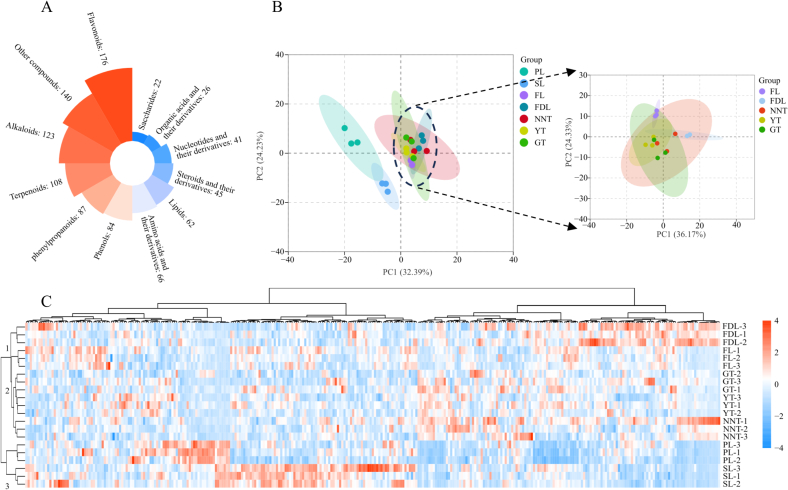
Analysis of nonvolatile metabolites. (A) Metabolic classes of nonvolatile metabolites. (B) PCA of the nonvolatile compounds during the processing of the three types of tea. (C) Hierarchical clustering of samples during the processing of the three types of tea.

In order to comprehensively understand the changes in substances during the processing of the three types of tea, samples were collected at each step and subjected to metabolomic detection. The curves of the total ion current from the MS analysis of the mixed QC samples showed that the retention time and peak intensity were consistent between samples, indicating the reliability of the detection method (Fig. S1 A and B). A total of 980 nonvolatile compounds from 12 classes were identified, including 176 flavonoids, 123 alkaloids, 108 terpenoids, 87 phenylpropanoids, 84 phenols, 66 amino acids and their derivatives, 62 lipids, 45 steroids and their derivatives, 41 nucleotides and their derivatives, 26 organic acids and their derivatives, 22 saccharides, and 140 other compounds (Fig. 4A). Although all the compounds were identified in each sample, their relative contents differed among samples.

Principal component analysis (PCA) of all the samples was used to understand the overall metabolic differences among samples (Fig. 4B). PCA1 explained 56.62 % of the variability and showed that the metabolites of PL and SL were different from those of FL, FDL, NNT, GT, and YT. This result indicated that the metabolites were not significantly altered by the end of the fixing step. PCA2 further explained 30.67 % of the variability, including the variability after the spreading step. It showed that FL was different from NNT, GT, and YT, indicating that the three types of tea formed their quality characteristics after the fixing step.

Hierarchical clustering (HC) was used to determine the relationships among samples taken throughout the tea processing (Fig. 4C). Samples from the same step were clustered together, while samples from different steps were far from each other. All the samples were grouped into three branches, displaying the metabolic changes during the processing of the three types of tea. This result is consistent with the PCA results, indicating that further differential analysis was feasible.

### 3.4 Characterization of the differential compounds during tea processing

To clarify the influence of tea processing on the non-volatile metabolites present in the landrace tea plant populations of Zhenfeng, 319 differential compounds of 12 classes were identified from the raw data (VIP ≥ 1 and *p* < 0.05). Flavonoids, amino acids and their derivatives, alkaloids, nucleotide and their derivatives, phenylpropanoids, and lipids comprised the largest number of differential metabolites.

**Fig. 5:**
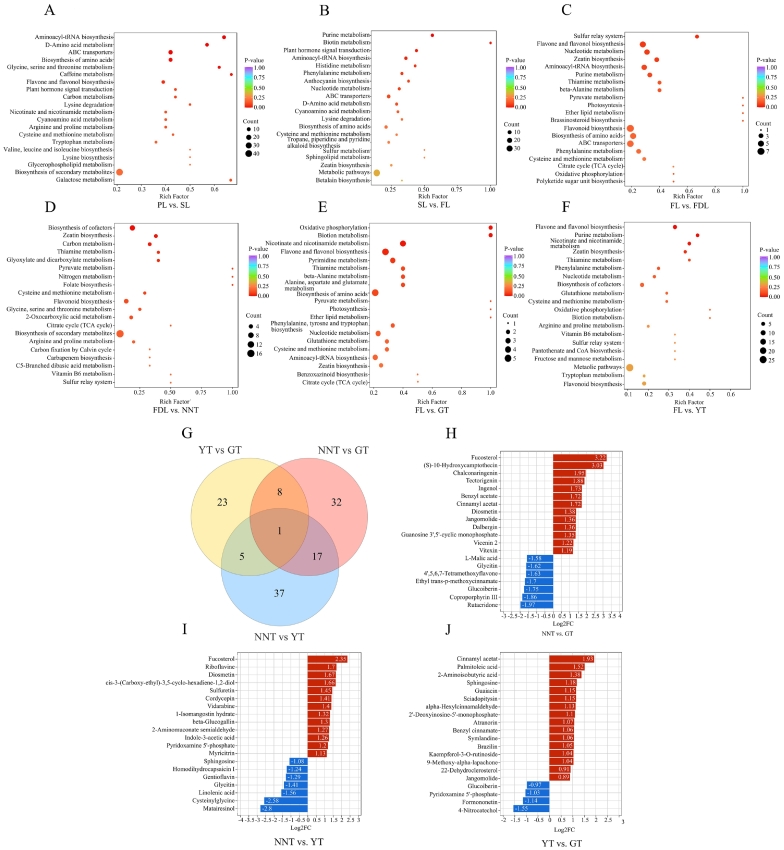
Differential compounds in tea processing. A-F, KEGG pathway enrichment of the differential compounds during processing. G, Venn diagram of differential compounds in the three types of tea. H-J, Bar chart of top fold changes in NNT vs. GT, NNT vs. YT, and YT vs. GT.

Kyoto Encyclopedia of Genes and Genomes (KEGG) analysis revealed the metabolic pathways enriched during the tea processing (Fig. 5A-F). The PL, SL, and FL samples were obtained from the common processing stages for NNT, GT, and YT. In PL vs. SL, the amino acid metabolism, caffeine metabolism, flavone and flavonol biosynthesis, and carbon metabolism pathways were enriched, while in SL vs. FL, the purine metabolism, amino acid metabolism, anthocyanin biosynthesis, and alkaloid biosynthesis pathways were enriched. These pathways involve the biosynthesis and decomposition of amino acids with taste properties, bitter purine akloids, bitter flavonoids and sweet sugars. However, samples taken after fixing were very different from each other due to the different final processing steps (direct drying for GT, yellowing and drying for YT, and twice bunch-drying for NNT). The FL vs. FDL, FL vs. GT, and FL vs. YT comparisons were enriched in similar pathways, such as the flavone and flavonol, nucleotide metabolism, zeatin biosynthesis, thiamine metabolism, purine metabolism, and biosynthesis of amino acids pathways. FDL vs. NNT was especially enriched in carbon metabolism, which involve sugar metabolism. Hence, the final processing stage (second bunch-drying) of NNT may be the most important factor causing it to taste differently from GT and YT.

A Venn diagram was used to compare the compounds related to the taste of NNT, GT, and YT (Fig. 5G). There were 58 (32 upregulated, and 26 downregulated), 60 (30 upregulated, and 30 downregulated), and 37 (27 upregulated, and 10 downregulated) differential compounds in the NNT vs. GT, NNT vs. YT, and YT vs. GT groups, respectively. The Venn diagram showed the common and specific differential compounds in the three pairwise comparisons. Jangomolide was a common differential compound, and 28, 29, and 30 specific differential compounds were identified in the three pairwise comparisons, respectively. The different taste attributes of the three types of tea are likely associated with these specific differential compounds, which were differentially accumulated during the last tea processing steps.

To further investigate the accumulation of non-volatile compounds in the three types of tea, we screened and compared the top differential compounds (TDCs) with the highest fold changes (Fig. 5H-J). In NNT vs. GT and NNT vs. YT, there were 13 TDCs upregulated and 7 TDCs downregulated, respectively. In YT vs. GT, there were 16 TDCs upregulated and 4 TDCs downregulated. These TDCs may be related to the differences in taste among NNT, GT, and YT.

### 3.5 Identification of the factors contributing to the sweet taste of NNT

**Fig. 6:**
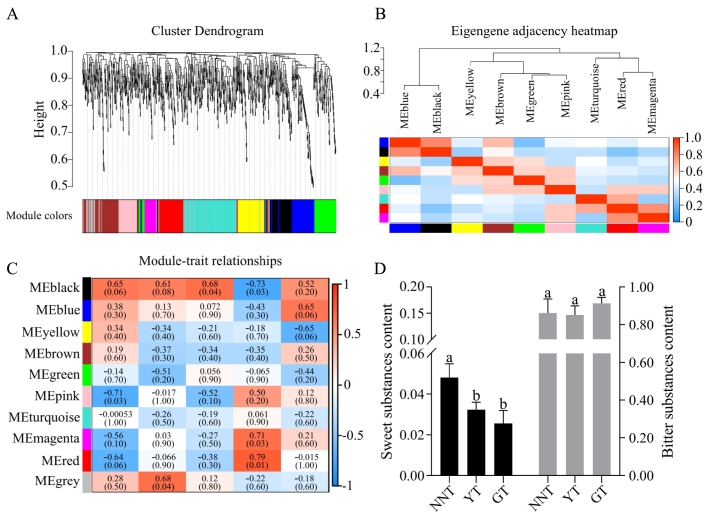
Correlations of metabolites with taste attributes based on WGCNA. (A) Clustering dendrogram of the average network adjacency for the identification of metabolite co-expression modules. (B) Eigengene adjacency heat map. (C) Module-trait relationships. (D) Bar chart of the content of sweet and bitter substances.

WGCNA was used to explore the factors related to the sweet taste by analyzing co-expression of the 980 chemical compounds and the strength of the five sensory attributes of the three types of tea. A total of 10 modules were obtained from the WGCNA and they were clustered into three groups (Fig. 6A, B). The black module in group 1 was significantly and negatively correlated with sweetness; the pink module in group 2 was significantly and negatively correlated with bitterness; and the magenta and red modules in group 3 were significantly and positively correlated with sweetness (|r| ≥ 0.7, *P* ≤ 0.05) (Fig. 6C). A total of 293 compounds were significantly correlated with sweetness and bitterness, 25 of which were significantly and differentially accumulated in NNT, GT, and YT (Table S1).

In order to further explore the important compounds related to tea sweetness, the differential compounds and quantitative description of the taste of the tea infusions were submitted to PCC analysis (|r| ≥ 0.7, *P* ≤ 0.05) (Fig. S2). Seven substances were significantly correlated with sweetness, and were consistent with the substances identified in WGCNA. Based on the current literature, another four compounds (theophylline, caffeine, L-leucine, 1-kestose and L-gulose) (Zeng et al., 2022; Zhou et al., 2019; https://cosylab.iiitd.edu.in/flavordb2/) that were previously identified with sweet or bitter attributes were found to be differentially accumulated in the three types of tea.

Thus, 329 substances from WGCNA, PPC, and the literature were considered to be associated with tea sweetness. Thirty compounds with taste attributes that were significantly and differentially accumulated in the three types of tea, including 20 for bitterness, eight for sweetness, and one for sourness, were further analyzed and found to be significantly correlated with sweetness (Table 1). The contents of substances that were associated with sweetness were significantly higher while those of substances that were associated with bitterness were significantly lower in NNT compared to in GT (Fig. 6D). These results indicated that the decrease in content of bitter compounds and the significant increase in content of sweet compounds may contribute to the sweetness of NNT.

**Table 1:** 30 key substances related to sweetness.

Compound name	Class	CAS	Taste	NNT	YT	GT
1-Kestose	Saccharides	470–69-9	Sweet	1.26 E-04 ± 4.34 E-05a	1.31 E-04 ± 3.01 E-05a	9.43 E-05 ± 9.58 E-05b
Mangiferin	Others	4773-96-0	Sweet	8.27 E-05 ± 7.61 E-06b	1.14 E-04 ± 7.19 E-06b	4.21 E-05 ± 2.65 E-05a
Indole-3-acetic acid	Organic acids and their derivatives	87–51-4	Sour	1.81 E-04 ± 2.05 E-05a	7.48 E-05 ± 2.87 E-05b	8.91 E-05 ± 3.98 E-05b
Homoeriodictyol	Flavonoids	446–71-9	Bitter	4.72 E-03 ± 3.96 E-03b	9.63 E-03 ± 1.43 E-04a	6.73 E-04 ± 1.27 E-04b
2′,3,5,7-Tetrahydroxyflavone	Flavonoids	480–15-9	Bitter	1.06 E-02 ± 1.83 E-03b	1.17 E-02 ± 9.32 E-04b	6.58 E-03 ± 1.78 E-03a
Thiamine	Others	59–43-8	Bitter	1.76 E-04 ± 1.32 E-05a	9.50 E-05 ± 2.92 E-06c	1.28 E-04 ± 9.26 E-06b
Myricetin	Flavonoids	529–44-2	Bitter	1.24 E-03 ± 1.97 E-04a	7.56 E-04 ± 4.47 E-05b	7.02 E-04 ± 7.29 E-05b
beta-Glucogallin	Phenols	13,405–60-2	Bitter	1.41 E-03 ± 2.10 E-04a	5.73 E-04 ± 1.88 E-04b	6.81 E-04 ± 2.39 E-04b
Theophylline	Alkaloids	58–55-9	Bitter	1.10 E-02 ± 9.44 E-03a	8.91 E-04 ± 1.09 E-04b	7.38 E-04 ± 1.63 E-04b
Fisetin	Flavonoids	528–48-3	Bitter	3.40 E-03 ± 8.44 E-04a	5.83 E-04 ± 3.49 E-04b	4.84 E-04 ± 2.21 E-04b
Pristimerin	Triterpenoids	1258-84-0	Bitter	4.33 E-05 ± 5.73 E-06a	3.48 E-05 ± 3.20 E-06ab	2.20 E-05 ± 1.43 E-05b
Benzyl acetate	Phenols	140–11-4	Bitter	3.27 E-04 ± 3.14 E-05a	1.96 E-04 ± 1.16 E-04ab	9.89 E-05 ± 9.72 E-06b
Kojibiose	Saccharides	2140-29-6	Sweet	3.90 E-03 ± 3.35 E-04a	2.38 E-03 ± 2.17 E-04b	2.43 E-03 ± 4.80 E-04b
Stachyose	Saccharides	10,094–58-3	Sweet	3.54 E-04 ± 2.60 E-05a	1.52 E-04 ± 1.32 E-04b	6.10 E-05 ± 1.02 E-05b
Plantagoside	Flavonoids	78,708–33-5	Sweet	1.22 E-04 ± 1.64 E-05a	5.25 E-05 ± 1.21 E-05b	4.75 E-05 ± 3.13 E-05b
Turanose	Saccharides	547–25-1	Sweet	4.35 E-02 ± 7.20 E-03a	2.95 E-02 ± 3.48 E-03b	2.26 E-02 ± 5.67 E-03b
trans-Caffeic acid	Phenylpropanoids	501–16-6	Bitter	7.66 E-04 ± 1.47 E-04a	1.25 E-03 ± 1.66 E-04b	1.39 E-03 ± 2.08 E-04b
trans-Hinokiresinol	Phenylpropanoids	17,676–24-3	Bitter	5.70 E-05 ± 1.51 E-05a	3.02 E-04 ± 1.11E-05b	3.20 E-04 ± 8.53 E-05b
Flavone	Flavonoids	525–82-6	Bitter	2.81 E-05 ± 4.86 E-07c	1.10 E-03 ± 2.97 E-05a	8.87 E-04 ± 2.46E-06b
L-Tryptophan	Amino acids and their derivatives	73–22-3	Bitter	2.66 E-04 ± 3.06 E-05b	3.33 E-04 ± 2.43 E-05a	2.92 E-04 ± 3.93 E-05ab
L-Leucine	Amino acids and their derivatives	73–32-5;61–90-5	Bitter	1.83 E-03 ± 3.59 E-04b	2.42 E-03 ± 3.81 E-04ab	2.89 E-03 ± 6.17 E-04a
Nobiletin	Flavonoids	478–01-3	Bitter	4.72 E-07 ± 3.33 E-08b	4.44 E-07 ± 1.35 E-08b	6.53 E-05 ± 2.13 E-05a
Linamarin	Others	554–35-8	Bitter	5.00 E-05 ± 3.56 E-05b	3.75 E-05 ± 4.60 E-06b	1.13 E-04 ± 7.45 E-06a
Ganoderic acid N	Triterpenoids	110,241–19-5	Bitter and sour	5.68 E-05 ± 2.09 E-05b	6.24 E-05 ± 8.97E-06b	9.24 E-05 ± 3.15E-06a
Isorhamnetin	Flavonoids	480–19-3	Bitter	5.69 E-05 ± 3.75 E-05b	1.11 E-04 ± 9.82 E-06ab	1.68 E-04 ± 4.96 E-05a
Formononetin	Flavonoids	485–72-3	Bitter	7.95 E-06 ± 2.82 E-06b	7.58 E-06 ± 3.58 E-06b	1.61 E-05 ± 3.63 E-06a
L-Phenylalanine	Amino acids and their derivatives	63–91-2	Bitter	1.14 E-01 ± 2.27 E-02b	1.17 E-01 ± 1.80 E-02ab	1.52 E-01 ± 1.14 E-02a
L-Gulose	Saccharides	6027-89-0	Sweet	6.76 E-05 ± 3.16 E-05c	8.28 E-05 ± 3.45 E-05b	1.11 E-04 ± 6.09 E-05a
Raffinose	Saccharides	512–69-6	Sweet	4.80 E-07 ± 2.58 E-08b	4.39 E-07 ± 8.15 E-09b	1.87 E-04 ± 5.14 E-05a
Caffeine	Alkaloids	58–08-2	Bitter	5.60 E+00 ± 3.64 E-01b	5.85 E+00 ± 1.92 E-01b	6.86 E+00 ± 8.21 E-02a

In order to explore the key substances that affected the sweetness of the three types of tea, the differential substances that were formed by drying, yellowing, and twice bunch-drying in the final processing of GT, YT, and NNT were further analyzed. Among the eight sweet substances, mangiferin and plantagoside are flavonoids, while the others are sugars. Turanose, kojibiose, stachyose, and plantagoside contents were significantly higher in NNT. L-Gulose and raffinose contents were highest in GT. 1-Kestose and mangiferin contents were highest in YT. Turanose and kojibiose had higher relative contents than other compounds, with a 10–100-fold change (FC), followed by stachyose. Compared to GT and YT, the contents of turanose, kojibiose, and stachyose in NNT increased by 1.92-FC and 1.47-FC, 1.60-FC and 1.64-FC, and 5.80-FC and 2.33-FC, respectively, indicating that the increase in these three sugars may be the key factor driving the sweetness of NNT. The 21 bitter compounds included eight flavonoids, three amino acids, three alkaloids, two terpenoids, two phenolic acids, and three other compounds. As 3,5,7,2’-Tetrahydroxyflavone, homoeriodictyol, fisetin, and myricetin, and flavone had higher relative contents (Table 1), these flavonoids may be important for the bitterness of tea. Three flavonoids, nobiletin, isorhamnetin, and formononetin, had significantly lower contents in NNT than in GT and YT, and they also contributed to the decrease in bitterness of the NNT infusion. Indole-3-acetic acid is a sour compound and ganoderic acid N has both bitter and sour tastes. These two compounds were significantly correlated with tea bitterness. Caffeine and theophylline contents in alkaloids were highest in NNT, and *L*-phenylalanine and L-leucine contents in amino acids, and β-glucogallin content in phenolic acids were lowest in NNT. The differential accumulation of these compounds may significantly affect tea sweetness.

### 3.6 Exploration of the key processing step for improving tea sweetness

**Fig. 7:**
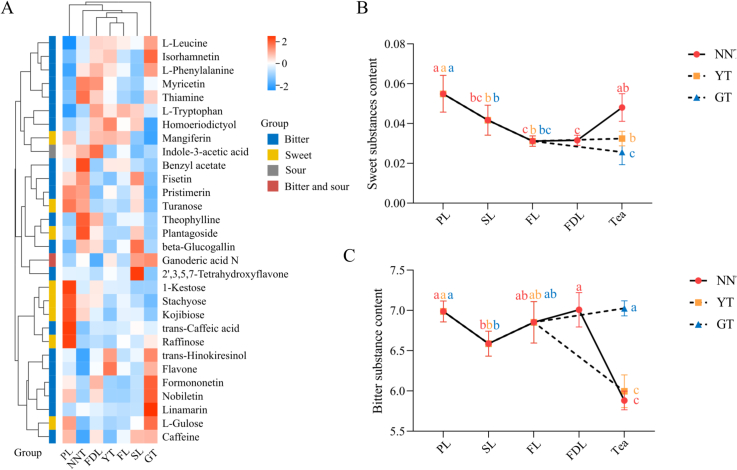
Analysis of 30 key substances. (A) HC analysis of the 30 substances and the content changes of key substances throughout processing. (B) Line chart showing changes in combined content of sweet substances during processing. (C) Line chart showing changes in total content of bitter substances during processing.

In order to comprehensively understand the changes in the substances related to sweetness during tea processing, the 30 key differential compounds were further analyzed using HC (Fig. 7A). The contents of key substances at every processing step were clustered into three groups in the HC analysis. Seven bitter substances were clustered into group 1, and them were highly accumulated in FDL. Five bitter substances in group 2 were significantly and highly accumulated in NNT compared to GT and YT. As for the sweet substances, five of eight were clustered into group 3. They were mostly accumulated in PL and then decreased throughout the tea processing. Two substances with a sour taste, indole-3-acetic acid and ganoderic acid N, were also analyzed as key substances. The former was accumulated more in NNT than in GT and YT, while the latter was accumulated more in GT, followed by YT, and least in NNT. The HC analysis revealed that the different sweetness levels of the three types of tea were likely correlated with the content changes of the 30 substances.

The overall accumulations of sweet and bitter substances throughout the processing were determined. The combined content of the eight differential sweet substances continuously decreased during the processing until the final processing step of GT and YT, and until the FDL sample of NNT; however, it significantly increased after the second bunch-drying (the final processing of NNT) (Fig. 7B). The combined content of the 21 differential bitter substances decreased after the spreading process and then began to increase continuously after the fixation process. However, the content of bitter substances in NNT significantly decreased after the second bunch-drying. These results indicated that the second bunch-drying was likely the key processing step for improving the sweetness of NNT (Fig. 7C).

## 4 Discussion

### 4.1 NNT processing enhances tea sweetness

Sweetness is an important quality index for elite tea. The contents and proportions of the main quality components strongly affect the taste of tea infusions. Teas that are made from varieties with high contents of total polyphenols and caffeine are often bitter and astringent, while those from varieties with high contents of free amino acids and soluble sugars often present sweet and umami tastes. In spring, tea leaves have the lowest levels of total polyphenols and the highest levels of free amino acids, but this trend is reversed in summer and autumn. In tea processing, fermenting and yellowing promote the oxidation of polyphenols, the degradation of proteins into small peptides and amino acids, and the degradation of insoluble polysaccharides into soluble sugars, thereby reducing the bitterness of the tea infusion and improving its freshness and sweetness (Y. Li et al., 2025; Xu et al., 2018). NNT is famous for its sweet and mellow taste and flowery aroma (Li, Peng, et al., 2022; J. Wei et al., 2025). It is produced from local tea plant varieties and is processed with two bunch-drying steps that are quite different from the final drying of GT and the yellowing step of YT. In order to understand whether the sweetness of NNT was related to the picking season or its unique processing method, three types of tea (NNT, GT, and YT) were made from the summer tea leaves of a local variety. The GT and YT were used as controls for taste and substance comparisons.

The results of the sensory quantitative description analysis of the three types of tea showed that NNT was the sweetest and the least bitter and astringent. GT had a strong bitter and astringent taste, while YT had moderate sweetness, bitterness, and astringency. The PCC analysis showed that soluble sugars and caffeine were the key contributors of the sweetness of the tea infusion, followed by total polyphenols. The main quality components, such as soluble sugars, caffeine, and total polyphenols, were considered to be related to the sweetness, bitterness, and astringency of the tea infusions, while free amino acids contents were considered to be related to the umami taste. These results indicated that the sweetness of NNT may be due to the unique processing method rather than the tea variety or the tea production season.

### 4.2 The second bunch-drying step is key for the accumulation of the sweet substances and degradation of bitter substances

The NNT processing method plays an important role in improving the sweetness of the tea infusion. In order to explore the key steps, differential compounds were analyzed during processing. The same raw materials, spreading conditions, and fixing conditions were used for GT, YT, and NNT. Therefore, although there were changes in taste contributors in the enriched pathways during these steps, such as amino acid metabolism, caffeine metabolism, flavonoids and flavonols, purine metabolism, anthocyanin metabolism, and alkaloid metabolism, these steps did not lead to differences in the sweetness among NNT, GT, and YT. In the final steps of the three types of tea, which responsible for quality formation of each tea, carbon metabolism was enriched in FDL vs. NNT, different from FL vs. FDL, FL vs. GT, and FL vs. YT (Fig. 3A). Carbon metabolism likely supplied sugars in the final bunch-drying of NNT. The accumulation of sweet and bitter substances changed substantially during the second bunch-drying (Fig. 5B). These results indicated that the second bunch-drying of NNT may be a key step in improving tea sweetness.

There were significant differences in tea compounds due to processing after the fixing step. The direct drying used for GT led to the retention of polyphenols and caffeine, while the yellowing step of YT and the two bunch-drying steps of NNT promoted the hydrolysis or decomposition of tea polyphenols as well as an increase in soluble sugars. During yellowing, various thermochemical reactions, such as nonenzymatic oxidation of polyphenols, decomposition of chlorophyll, and protein hydrolysis, change the composition of the tea, giving YT a sweet and umami taste (Y. Wei et al., 2021). The bunch-drying of NNT creates a similar humid environment to yellowing. It is speculated that the external moisture of tea leaves evaporates during the first bunch-drying. After this, the internal moisture in tea permeates to the outside and evaporates during the second bunch-drying. The twice bunch-drying process is therefore similar to conducting yellowing twice, which reduces bitterness and increases sweetness (Y. Wei et al., 2020). This may explain why NNT is sweeter than YT and GT.

### 4.3 Changes in the key taste contributors and the mechanisms affecting tea sweetness

A total of 30 differential substances were identified with sweet (eight substances), bitter (21 substances), or sour (one substance) tastes. Six sugars (turanose, kojibiose, stachyose, 1-kestose, L-gulose, and raffinose), mangiferin, and plantagoside were identified as sweet substances. As contents of turanose and kojibiose were highest in the three types of tea and significantly higher in NNT, these two disaccharides were further analyzed and are discussed here. Turanose is a structural isomer of sucrose that naturally exists in honey, pollen, and sugarcane, with about 50 % of the sweetness of sucrose (Park et al., 2016). It inhibits lipid accumulation and thus can control obesity (Park et al., 2016). The biosynthesis of turanose in plants involves various enzymes, namely transglucosylase, cyclomaltodextrin glucanotransferase, sucrose isomerase, trehalose synthase, amylosucrase, dextransucrase, and branching sucrase (Gangoiti et al., 2018). In vitro, turanose can be hydrolyzed into glucose and fructose under the action of α-glucosidase (Tewari & Goldberg, 1991), and also can be produced by isomerization from sucrose under thermal conditions (Farah, 2019). In this study, turanose levels were low in the SL, FL, FDL, GT, and YT samples; however, they were high in NNT. This indicates that the hydrolysis of turanose was stronger than its biosynthesis during plucking, fixation, and the first drying step, but isomerization increased under the lower temperature and shorter time during the second bunch-drying step of NNT processing. Kojibiose is a natural disaccharide that can be found in the hydrolysate of starch (Sato & Aso, 1957). Kojibiose hydrolysis and decomposition are temperature-dependent: the first at lower temperatures represents long chain scission, and the second at higher temperatures involves decomposition of glucose ring (X. Liu et al., 2008, Liu et al., 2013). In our study, PL had the highest content of kojibiose, then SL, FL and FDL, with GT and YT having the lowest contents. However, the kojibiose content was significantly increased in NNT. We speculate that long chain scission under the relatively lower temperature during the second bunch-drying may have played a role in the formation of kojibiose. Stachyose, 1-kestose, mangiferin, and plantagoside contents were also higher in NNT than in GT and YT. Stachyose can maintain body health by inhibiting inflammation and regulating lipid metabolism (Jiang et al., 2023). 1-Kestose can improve the intestinal environment and activate the gut immune system (Shimomura et al., 2017). Mangiferin has diverse pharmacological activities, including anti-inflammatory, neuroprotective, anti-diabetic, immunomodulatory, and anti-cancer activities (Ashok et al., 2021). Plantagoside inhibits protein cross-linking glycation and has potential beneficial applications in preventing complications of diabetes (Matsuura et al., 2014). The significant increase of these saccharides enhanced the sweetness, on the other hand, improved the healthy function of NNT.

Out of the 21 bitter substances, 10 had higher relative contents in NNT than in GT and YT, while 11 had significantly lower contents in NNT. Five flavonoids (2′,3,5,7-tetrahydroxyflavone, homoeriodictyol, fisetin, myricetin, and flavone), two alkaloids (caffeine and theophylline), two amino acids (*L*-phenylalanine and L-leucine), one phenol (β-glucogallinare), and one phenylpropanoid (trans-caffeic acid) were bitter substances with relative high contents in the three types of tea, and they were speculated to be important to tea bitterness. Flavonoids are natural, safe, and effective components found in fruits and vegetables. They have biological effects such as antibacterial, antioxidant, anti-inflammatory, antitumor, anticardiovascular, and neuroprotective activities, and they can delay immune aging. All five bitter flavonoids are aglycones. Aglycones are the hydrolyzation products of flavonoid glycosides and the main substances that are absorbed along the digestive tract. Hydrothermal treatment has been found to favor the formation of aglycones via the hydrolyzation of flavonoid glycosides and the conversion of β-glucoside isoflavones, while baking promotes aglycone degradation via oxidation and breaking of the rings in their molecular structure (Sanches de Lima & Ida, 2014). In the current study, spreading increased the contents of most aglycones except for flavone, which may be related to the hydrolysis of flavonoids and conversion of isoflavonoids. The fixing and drying of GT reduced the contents of 2′,3,5,7-tetrahydroxyflavone, homoeriodictyol, and fisetin, likely due to the degradation of aglycones under thermal conditions. However, the twice bunch-drying of NNT and yellowing of YT significantly increased their levels, which is likely related to the hydrolysis of flavonoid glycosides and the conversion of isoflavones. Thermal treatment was reported to favor the conversion of dihydromyricetin to myricetin and to accelerate the degradation of myricetin (W.-N. Liu & Zhao, 2019; B. Zhou et al., 2018). Myricetin could accelerate the oxidation of bitter and astringent EGCG during storage and cause tea infusion browning (Q. Dai et al., 2017) and a reduction in bitterness and astringency. However, the level of myricetin was increased in this study, and the level in NNT was significantly higher than that in YT and GT. It is speculated that the hydrolysis reaction of myricetin glycosides is greater than the degradation of myricetin aglycone during drying, yellowing, and especially the twice bunch-drying. Flavone contents showed the opposite trend compared with the contents of the above four flavonoids; flavone increased significantly in GT and YT but decreased significantly in NNT. It is speculated that thermal conditions accelerate the hydrolysis of flavonoids into flavone, but long-term thermal conditions caused the degradation of flavone. As for the other flavonoids, most of them decreased significantly in NNT compared to GT and YT, reducing the bitterness and astringency of NNT. However, their contents were quite low and therefore they were considered less important for the flavor of the three types of tea.

β-Glucogallin, a plant-derived polyphenolic ester, is regarded as the primary metabolite in the biosynthesis of hydrolyzable tannins. β-Glucogallin has pharmacological activities, such as antioxidant, anti-inflammatory, antidiabetic, cataract-preventing, antiglaucoma, and UV-protectant activities (Khan et al., 2022). During the processing of the three types of tea, β-glucogallin increased in the spreading stage, potentially due to the combination of uridine diphosphate (UDP)-glucose and gallic acid (Yang et al., 2024). β-Glucogallin decreased during fixing and drying of YT and GT, likely due to hydrolyzation. *Trans*-Caffeic acid has excellent antioxidant properties and is prone to be isomerized into cis-caffeic acid (Razboršek et al., 2021) and even broken into phenols (Nastasiienko et al., 2021) as a phenolic acid when exposed to thermal stress. In this study, the trans-caffeic acid decreased in all the samples, especially in NNT. The isomer cis-caffeic acid increased significantly in the FL, FDL, and GT samples, likely due to the isomerization of trans-caffeic acid. *Cis*-Caffeic acid decreased significantly in YT and NNT, which may be related to the degradation of decarboxylation.

Caffeine and theophylline are important purine alkaloids and are involved in the inter-conversion in purine metabolism in tea plants. The fermentation of black and dark tea increases the contents of alkaloids such as caffeine and theophylline, and microorganisms in dark tea demethylate caffeine and convert it into theophylline (Y. Wang, Kan, et al., 2019) (B. Zhou et al., 2020). In our study, the content of caffeine was reduced during all the processing stages of the three types of tea, and it was significantly lower in NNT than in GT and YT, consistent with previous research results (Y. Wang, Kan, et al., 2019). Theophylline was significantly decreased in GT and YT, but substantially increased after the twice bunch-drying in the NNT process, suggesting that twice bunch-drying favors the conversion of caffeine into theophylline by the demethylation reaction. Given that the content of caffeine was much higher than that of theophylline in the three types of tea, it is believed that a significant reduction in caffeine is more critical.

Amino acids are important components for the taste of tea, imparting a fresh and refreshing taste that can harmonize the bitterness and astringency of tea infusions. During the withering and fixing stages, the total amino acid content significantly increases due to biosynthesis and stress-induced proteolysis, whereas the drying stage significantly decreases the amino acid content through Maillard and Strecker reactions (Yu, Zhang, et al., 2023). In this study, the analysis of the main quality components showed that the total amino acid content was not significantly correlated with the sweetness of the tea infusions (Fig. 1B). L-Phenylalanine and L-leucine were significantly and negatively correlated with the sweetness of tea infusion due to their bitter attribution. During processing of the three types of tea, *L*-phenylalanine and L-leucine generally increased. However, in NNT processing, they increased during the first bunch-drying but significantly decreased during the second bunch-drying, which caused NNT to have lower levels compared to GT and YT. The differential accumulation of these two amino acids in NNT, GT, and YT is likely due to the different rates of proteolysis and Maillard and Strecker reactions in the different conditions of the last stages of tea processing.

**Fig. 8:**
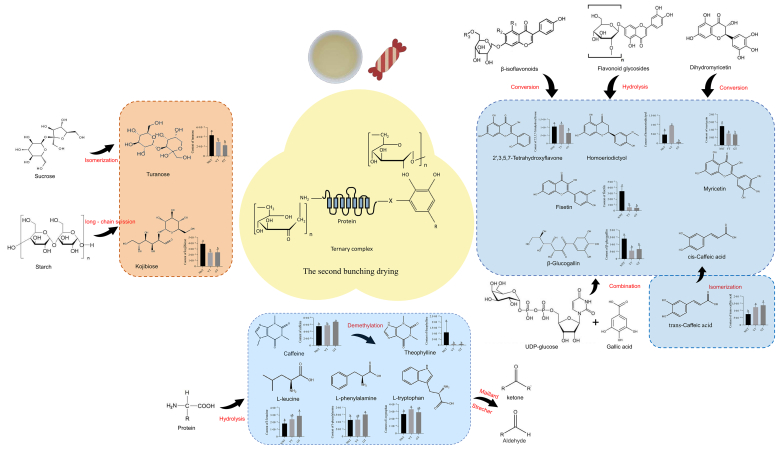
The mechanism of improving sweetness in NNT infusion. The light orange-colored area represents sweet-tasting substances; The light blue-colored area represents bitter-tasting substances; The yellow-colored area represents ternary complexes. (For interpretation of the references to color in this figure legend, the reader is referred to the web version of this article.)

Piezo2 is considered a mechanoreceptor for the human perception of astringency (Moayedi et al., 2018). Piezo2 can be activated by the interaction between phenols and mucin or proline-rich proteins (F. Wei, Wang, et al., 2023). The addition of saccharides may combine into ternary complexes, polysaccharide-polyphenol-protein, through hydrophobic interactions, hydrogen bonds, and other non-covalent bonds (Y. Zhang et al., 2022). The ternary complexes can inhibit astringency by increasing water solubility, steric hindrance, and electrostatic repulsion. In tea infusions, the substantial phenols, including flavonoids and phenolic acids, provide a recombination taste as bitterness and astringency. Therefore, although the contents of flavonoids and phenolic acids were significantly higher in YT and NNT than in GT, the bitterness and astringency were less intense. Thus, the significantly increased sugars and the significantly decreased amino acids and caffeine were speculated to play a decisive function in improving the sweetness of NNT (Fig. 8).

### 4.4 Potential application of the NNT process to improve the quality of summer and autumn tea

Tea planting areas and tea production in China are increasing; however, the utilization rate of fresh tea leaves growing in summer and autumn is low. Summer and autumn leaves have a high maturity and significantly higher contents of total polyphenols and caffeine, giving the tea a pronounced bitter and astringent taste. As a result, thousands of tons of summer and autumn tea leaves are discarded every year (Liang et al., 2024). The unique twice bunch-drying process of NNT provides a new idea for solving this problem; it could be used to regulate the factors that contribute to sweetness. Twice bunch-drying is similar to conducting yellowing twice. The specific temperature and humidity conditions of bunch-drying resulted in thermochemical reactions that remarkably decreased the content of bitter caffeine but retained flavonoids. Concurrently, it elevated the concentration of soluble sugars, thereby effectively enhancing the sweetness of the final tea infusion. In this study, the second bunch-drying was more crucial for the formation of sweetness in the tea infusion. Therefore, we speculate that twice bunch-drying could be used to improve the taste of summer and autumn tea by retaining more flavonoids, significantly increasing sugars, and decreasing bitter alkaloids. Further research is needed to focus on optimizing the parameters for the second bunch-drying stage, such as the moisture content of the material, drying time, and drying temperature. An optimized NNT process has great potential to improve the quality of summer and autumn tea.

## 5 Conclusions

This study screened 30 important substances related to NNT sweetness through sensory evaluation, non-targeted metabolomics analysis, PCC analysis, and a literature search, and explored changes in these substances during processing. The results showed that sugar substances, especially turanese and kojibiose, increased during processing, while the bitter-tasting L-phenyalanine and L-leucine, as well as caffeine, decreased. These components were fundamental for the sweetness of NNT. This study elucidated the mechanisms and key processing stage underlying the sweetness of NNT, providing a scientific basis for potential optimization of summer and autumn tea processing parameters and the development of low-bitter tea products.

## CRediT authorship contribution statement

**Qi Wei:** Writing – original draft, Software, Methodology, Investigation. **Xiaobo Liu:** Validation, Formal analysis. **Shanshan Long:** Methodology, Investigation. **Luqin Zhu:** Validation, Software, Methodology, Data curation. **Hongzhiyuan Yang:** Validation, Formal analysis. **Xue Song:** Methodology, Investigation. **Yishu Peng:** Methodology, Investigation. **Taolin Chen:** Validation, Formal analysis. **Jianjun Liu:** Validation, Investigation. **Beibei Wen:** Methodology, Investigation. **Meifeng Li:** Writing – review & editing, Project administration, Methodology, Investigation.

## Declaration of competing interest

The authors declare that they have no known competing financial interests or personal relationships that could have appeared to influence the work reported in this paper.

Funding

This work was supported by the 10.13039/100006190National Key Research and Development Plan [grant numbers 2022YFD1600801]; 10.13039/501100001809The National Natural Science Foundation of China [grant number 32260786; 32460783]; The Scientific Research Program of Qiannan Prefecture [grant number [2023] 12] The Guizhou Provincial Basic Research Program [grant number ZK[2022]-YB115] and 10.13039/501100003459The Basic Research Program of Guizhou University [grant number GDJC[2023]05)].10.13039/501100003459The Laboratory Open Project of Guizhou University [grant number SYSKF2024–020].

## Data Availability

Data will be made available on request.

## Appendix A Supplementary data

Supplementary material Image 1
