# Supplementary material for: Exploration of the substances and key processing steps related to the sweetness of Niangniang tea
Source: Food Chem X. 2025 May 15;28:102556. doi: 10.1016/j.fochx.2025.102556 (PMC12148608; doi:10.1016/j.fochx.2025.102556)
Supplement: Supplementary file 1 — Supplementary material [file mmc1.docx]

Table S1 25 compounds significantly correlated with sweetness or bitterness in WGCNA

| Compound name | CAS | Taste | NNT | YT | GT |
| --- | --- | --- | --- | --- | --- |
| Formononetin | 485-72-3 | Bitter | 7.95 E-06 ± 2.82 E-06b | 7.58 E-06 ± 3.58 E-06b | 1.61 E-05 ± 3.63 E-06a |
| Homoeriodictyol | 446-71-9 | Bitter | 4.72 E-03 ± 3.96 E-03b | 9.63 E-03 ± 1.43 E-04a | 6.73 E-04 ± 1.27E-04b |
| L-Phenylalanine | 63-91-2 | Bitter | 1.14 E-01 ± 2.27 E-02b | 1.17 E-01 ± 1.80 E-02ab | 1.52 E-01 ± 1.14 E-02a |
| Nobiletin | 478-01-3 | Bitter | 4.72 E-07 ± 3.33 E-08b | 4.44 E-07 ± 1.35 E-08b | 6.53 E-05 ± 2.13 E-05a |
| trans-Caffeic acid | 501-16-6 | Bitter | 7.66 E-04 ± 1.47 E-04b | 1.25 E-03 ± 1.66 E-04a | 1.39 E-03 ± 2.08 E-04a |
| Benzyl acetate | 140-11-4 | Bitter | 3.27 E-04 ± 3.14 E-05a | 1.96 E-04 ± 1.16 E-04ab | 9.89 E-05 ± 9.72 E-06b |
| beta-Glucogallin | 13405-60-2 | Bitter | 1.41 E-03 ± 2.10 E-04a | 5.73 E-04 ± 1.88 E-04b | 6.81 E-04 ± 2.39 E-04b |
| Fisetin | 528-48-3 | Bitter | 3.40 E-03 ± 8.44 E-04a | 5.83 E-04 ± 3.49 E-04b | 4.84 E-04 ± 2.21 E-04b |
| Flavone | 525-82-6 | Bitter | 2.81 E-05 ± 4.86 E-07c | 1.10 E-03 ± 2.97 E-05a | 8.87 E-04 ± 2.46 E-06b |
| L-Tryptophan | 73-22-3 | Bitter | 2.66 E-04 ± 3.06 E-05b | 3.33 E-04 ± 2.43 E-05a | 2.92 E-04 ± 3.93 E-05ab |
| Myricetin | 529-44-2 | Bitter | 1.24 E-03 ± 1.97 E-04a | 7.56 E-04 ± 4.47 E-05b | 7.02 E-04 ± 7.29 E-05b |
| Thiamine | 59-43-8 | Bitter | 1.76 E-04 ± 1.32 E-05a | 9.50 E-05 ± 2.92 E-06c | 1.28 E-04 ± 9.26 E-06b |
| 2',3,5,7-Tetrahydroxyflavone | 480-15-9 | Bitter | 1.06 E-02 ± 1.83 E-03a | 1.17 E-02 ± 9.32 E-04a | 6.58 E-03 ± 1.78 E-03b |
| Linamarin | 554-35-8 | Bitter | 5.00 E-05 ± 3.56 E-05b | 3.75 E-05 ± 4.60 E-06b | 1.13 E-04 ± 7.45 E-06a |
| Pristimerin | 1258-84-0 | Bitter | 4.33 E-05 ± 5.73 E-06a | 3.48 E-05 ± 3.20 E-06ab | 2.20 E-05 ± 1.43 E-05b |
| Isorhamnetin | 480-19-3 | Bitter | 5.69 E-05 ± 3.75 E-05b | 1.11 E-04 ± 9.82 E-06ab | 1.68 E-04 ± 4.96 E-05a |
| trans-Hinokiresinol | 17676-24-3 | Bitter | 5.70 E-05 ± 1.51 E-05b | 3.02 E-04 ± 1.11 E-05a | 3.20 E-04 ± 8.53 E-05a |
| Ganoderic acid N | 110241-19-5 | Bitter and sour | 5.68 E-05 ± 2.09 E-05b | 6.24 E-05 ± 8.97 E-06b | 9.24 E-05 ± 3.15 E-06a |
| Indole-3-acetic acid | 87-51-4 | Sour | 1.81 E-04 ± 2.05 E-05a | 7.48 E-05 ± 2.87 E-05b | 8.91 E-05 ± 3.98 E-05b |
| Raffinose | 512-69-6 | Sweet | 4.80 E-07 ± 2.58 E-08b | 4.39 E-07 ± 8.15 E-09b | 1.87 E-04 ± 5.14 E-05a |
| Kojibiose | 2140-29-6 | Sweet | 3.90 E-03 ± 3.35 E-04a | 2.38 E-03 ± 2.17 E-04b | 2.43 E-03 ± 4.80 E-04b |
| Plantagoside | 78708-33-5 | Sweet | 1.22 E-04 ± 1.64 E-05a | 5.25 E-05 ± 1.21 E-05b | 4.75 E-05 ± 3.13 E-05b |
| Turanose | 547-25-1 | Sweet | 4.35 E-02 ± 7.20 E-03a | 2.95 E-02 ± 3.48 E-03b | 2.26 E-02 ± 5.67 E-03b |
| Mangiferin | 4773-96-0 | Sweet | 8.27 E-05 ± 7.61 E-06a | 1.14 E-04 ± 7.19 E-06a | 4.21 E-05 ± 2.65 E-05b |
| Stachyose | 10094-58-3 | Sweet | 3.54 E-04 ± 2.60 E-05a | 1.52 E-04 ± 1.32 E-04b | 6.10 E-05 ± 1.02 E-05b |


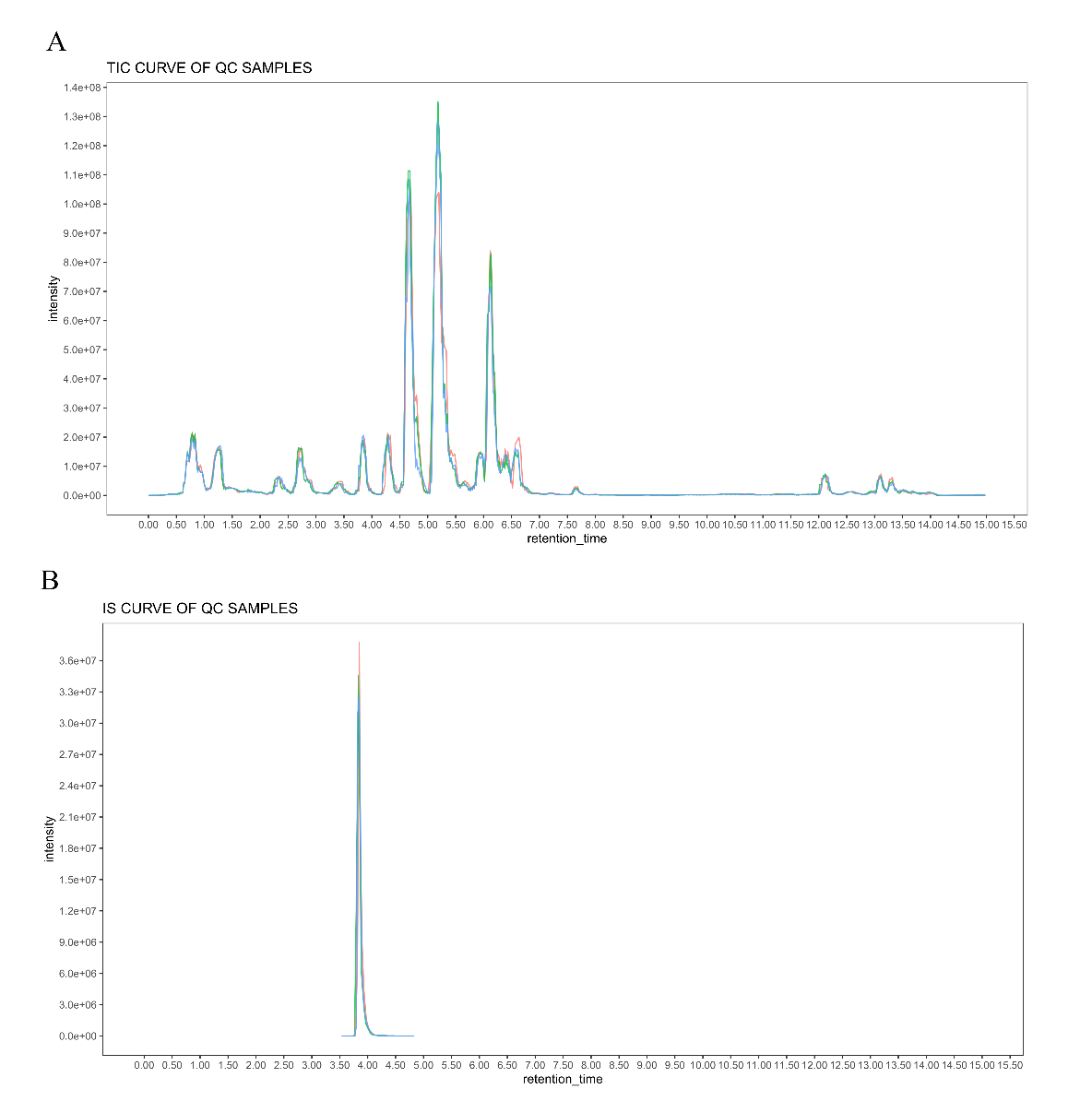


Fig. S1 Quality control measures (n=3) (A) Instrument stability monitoring, (B) Internal standard response. The abscissa represents the retention time, which is the time it takes for a compound to elute from the chromatographic column and reach the detector starting from the injection. It is used for qualitative analysis. Different compounds typically have specific retention times under the same chromatographic conditions. The ordinate represents the signal intensity, which reflects the content or abundance of the detected compound. The higher the signal intensity, the relatively larger the amount of the corresponding compound usually is.


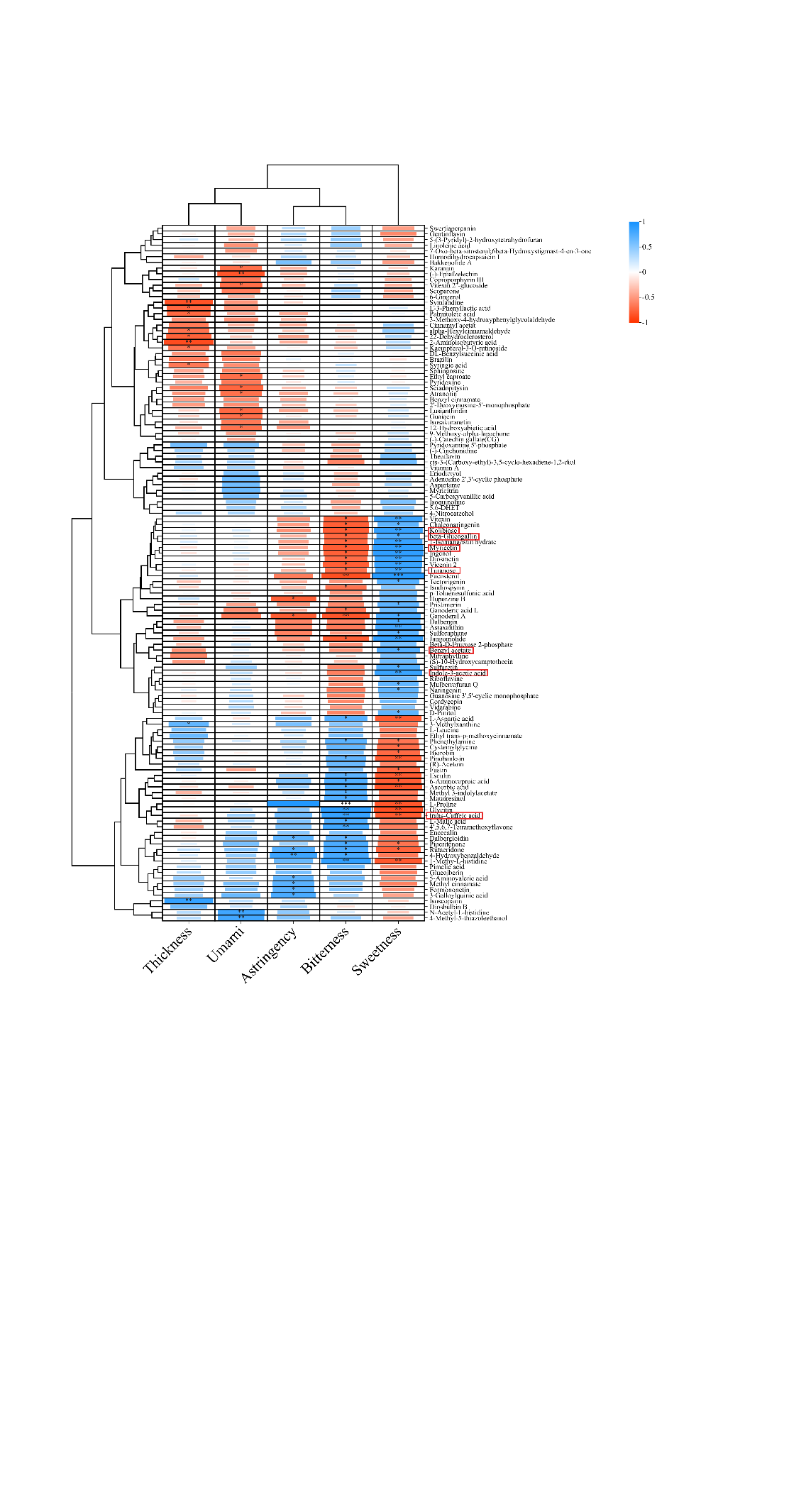


Fig. S2 Heat map of the correlation between differential compounds and quantitative description analysis. The abscissa represents the taste attributes of the tea infusion, and the ordinate represents the differential substances. In the visualization of the correlation, blue represents a negative correlation and red represents a positive correlation. The size of the internal columns reflects the degree of correlation. Ns indicates *p*>0.05, * indicates *p*<0.05, ** indicates *p*<0.01, *** indicates *p*<0.001.
